# Phenotypic and genotypic detection of extended spectrum β-lactamases among *Escherichia coli* and *Klebsiella pneumoniae* isolates from type 2 diabetic patients with urinary tract infections

**DOI:** 10.4314/ahs.v21i2.3

**Published:** 2021-06

**Authors:** Souad Youssouf Kani Elmi, Medhat Saber Ashour, Fathy Zakaria Alsewy, Nashwa Fawzy Abd El Moez Azzam

**Affiliations:** 1 Master Degree in Public Health, High Institute of Public Health, Alexandria University. Egypt. Bachelor's Degree in Medical Laboratories, Faculty of Medicine and Health Sciences, Sana'a University. Yemen; 2 High Institute of Public Health, Alexandria University. Egypt; 3 Department of Diabetes and Metabolism, Faculty of Medicine, Alexandria University. Egypt

**Keywords:** Extended Spectrum β-Lactamases, Type 2 diabetes mellitus, Urinary tract infections, Phenotypic and genotypic methods

## Abstract

**Background:**

T2DM patients are more likely to have UTIs caused by resistant organisms such as ESBLs producing bacteria. Challenging reliable identification and prompt characterization of in-vitro susceptibilities of these bacteria are the first steps of deciding the appropriate antimicrobial therapy for UTIs caused by them.

**Objectives:**

To isolate and identify *E. coli* and *K. pneumoniae* from urine of T2DM patients with UTIs, to determine antibiotic resistance pattern among isolates, and to identify ESBLs production phenotypically and genotypically.

**Material and method:**

All samples were cultured on Cystine-Lactose-Electrolyte-Deficient Agar medium (CLED) by using calibrated loop. Growth of 100 colonies or more, i.e. 105 colony forming units (CFU)/mL urine was considered as significant bacteriuria. Isolation and identification were done according to standard method. All isolates were tested for antibiotic susceptibility testing by the disc diffusion method according to CLSI guidelines. Phenotypic detection of ESBLs was done by double-disk synergy test. Genotypic detection of blaTEM, blaSHV and blaCTX-M genes by using PCR.

**Results:**

Results of this study showed that *E. coli* and *K. pneumoniae* were the dominant bacterial isolates, they constituted 103 (91.2%) out of 113 urine isolates. *E. coli* (58. 4%) *K. pneumoniae* (32.7%), *Enterococcus spp.* (4.4%), *Proteus spp.* (2.7%) and Pseudomonas spp. (1.8%). About 25 (24.3%) out of 103 E. coli and K. pneumoniae isolates were ESBLs positive by DDST, and 22 (88.0%) out of them had ESBLs encoding genes by conventional PCR. The most common gene detected was blaTEM (59.1%), followed by blaSHV (27.3%). CTX-M had not been detected in any of testes isolates.

**Conclusion:**

blaTEM and blaSHV genes were detected in 22 out of 25 ESBLs producing *E. coli* and *K. pneumoniae* isolates phenotypically detected by DDST. blaTEM was found to be the predominant gene (59.1%), while blaCTX-Mene was not detected in any of tested isolates.

## Introduction

Type 2 diabetes mellitus (T2 DM) is a metabolic disorder characterized by variable degrees of insulin resistance, impaired insulin secretion, and increased glucose production^1^. Patients with T2DM are at increased risk for urinary tract infections (UTIs) due to diabetic neuropathy that causes dysfunctional voiding and urinary retention, thus facilitating bacterial growth. High glucose content of urine provide nutrition for urinary microbes^2^. Patients with diabetes are more likely to have resistant organisms causing UTIs, including extended spectrum β-lactamases(ESBLs), carbapenem-resistant Enterobacteriaceae^3^–^5^. Based on the synergy between a third-generation cephalosporin and clavulanate, several phenotypic detection methods of ESBLs had been designed; such as double-disk synergy test (DDST)^6^. Alternative strategies to replace traditional phenotypic methods had been proposed; the most widely used techniques are conventional polymerase chain reaction (PCR) and gene sequencing^7^,^8^.

## Material and methods

### Study Area

The study was carried out over a period of four months from May 2019 to August 2019. It enrolled 113 type 2 diabetic patients suffering from UTIs attending the outpatient clinics of the Department of Diabetes and Metabolism or Department of Urology at Alexandria Main University Hospital. Alexandria city. Egypt.

### Laboratory investigation

#### Sampling

Clean catch midstream urine samples were aseptically collected from 113 type 2 diabetic patients with UTIs. Media-Test Combi10 reagent dipsticks were used for chemical urine analysis. They contain up to 10 different chemical pads or reagents (blood, urobilinogen, bilirubin, protein, nitrate, ketones, glucose, pH, specific gravity and leukocytes).

Semi-quantitative urine culture on CLED agar and biochemical identification of bacterial isolates were done9. All E. coli and K. pneumoniae isolates were tested for their antibiotic susceptibility using the Kirby Bauer disc diffusion method. Antibiotics used were Ampiillin, Cefaclor, Cefotaxime, Ceftazidime, Cefepime, Amoxicillin-clavulanic acid, Aztreonam, Imipenem, Amikacin, Norfloxacin and Nitrofurantoin. Inhibition zones were measured, and then susceptibility was recorded as susceptible (S), intermediate (I) and resistant (R) according to CLSI breakpoints.

### Double-disc synergy test

A lawn culture of each *E. coli* and *K. pneumoniae* isolates was made on MHA plate, as recommended by CLSI. A disc of amoxicillin-clavulanic acid (20/10 µg) was placed in the center of the plate at 20mm distance to ceftazidime (CAZ 30µg) and cefotaxime (CTX 30µg). ESBLs production was detected by the appearance of keyhole effect due to the enhanced activity of ceftazidime and cefotaxime with clavulanic acid^10^.

### Genotypic method for detection of ESBLs Producing *E. coli* and *K. pneumoniae* by conventional Polymerase Chain Reaction (PCR)

Genomic DNA was extracted using a DNA easy tissue kit (Qiagen, Germany) according to the instructions of the manufacturer.

### DNA Amplification protocol

Initial denaturation (94 °C for 5 min); 30 cycles of denaturation (94 °C for 30 s), annealing (58°C for 1 min) and polymerization (72 °C for 1 min); and an additional polymerization step (72 °C for 7 min)^11^.

### DNA detection

The amplification products were analysed by agarose gel electrophoresis and ethidium bromide staining^12^. The gel running plate was placed in its special gel casting plate supplied with electrophoresis apparatus (Mupid-Exu submarine electrophoresis system, Advance, Japan)

## Results

The majority of T2DM patients with UTIs were females (69%). Most of female patients, (47.4%) were of age group 55–65 years. 97.3% of cultured clean catch midstream urine samples yield significant bacteriuria, where 70% were cultured from female patients, and 30% from male patients. Glucose concentration in urine ws found to significantly affect the colony count of urine cultures, where (80.9%) urine samples with colony count ≥105 CFU/ml were cultured from T2DM patients with +3 glucosuria level (≥ 500mg\dl). *E. coli* and *K. pneumoniae* isolates were the most prevalent uropathogens, they had been isolated from 103 (91.2 %) out of 113 UTIs in T2DM. table (1)

**Table 1:** Common etiologic agents of UTIs in T2DM patients

Etiologic agents	No. (%)
*E. coli*	66 (58.4%)
*K. pneumoniae*	37 (32.7%)
*Enterococci*	5 (4.4%)
*Pseudomonas aeruginosa*	2 (1.8%)
*Proteus spp*	3 (2.7%)
**Total**	**113 (100%)**

About 24.3% of UTIs caused by E. coli and K. pneumoniae isolates were due to ESBLs-producing strains. The highest percentage of ESBL-UTIs (68%) were found in age groups of 55–65 years, and 80% of them were females. Duration of diabetes and poor glycemic control were not found to significantly increase the risk of ESBLs, however use of urinary catheter and prior use of antibiotics were found to be associated with ESBLs positivity. ESBLs production appeared as a significant risk factor for acute kidney injury.

**Figure 2:**
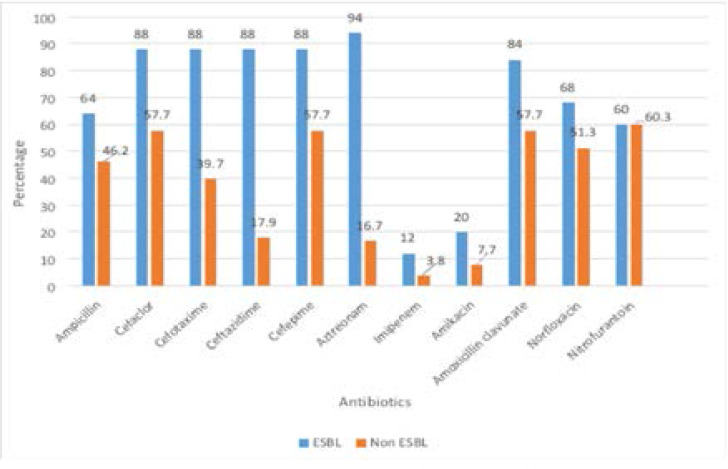
Antibiotics resitance patterns of ESBL and non ESBNL isolates

ESBLs producing isolates showed higher resistance rates to antibiotics than non ESBLs producing, this increase was significant with aztreonam, all cephalosporin, and amoxicillin clavulanate. Figure (2)

ESBLs genes were detected in only 22 out of 25 ESBLs producing isolates detected by DDST phenotypic method.

Three isolates (one *E. coli* and two *K. pneumoniae* isolates) were not confirmed by PCR to have ESBLs encoding genes.

The predominant gene detected was blaTEM, as it was detected in 13 (59.1 %) out of 22 confirmed ESBLs producing isolates. blaSHV was detected in 6 (27.3%) out of 22 confirmed ESBLs producing isolates. Three isolates harbored both genes (13.6 %). blaCTX was not detected in any of ESBLs producing isolates. Table (3)

**Table 3:** Prevalence of ESBLs genes among ESBLs producing isolates

ESBLs genes	Isolates		Total
	*E. coli (n=16)*	*K. pneumoniae (n=16)*	
	No. (%)	No. (%)	No. (%)
**TEM**	11(68.8%)	2 (33.3%)	13 (59.1%)
**SHV**	3 (18.7%)	3 (50%)	6 (27.3%)
**TEM+SHV**	2 (12.5%)	1(16.7%)	3 (13.6%)
**Total**	16(100%)	6 (100%)	22 (100%)

## Discussion

T2DM is a reported risk factor for frequent and severe UTIs due to depressed immunity, altered phagocytic adhesion and abnormal physiological process. Wide use of broad- spectrum antibiotics to treat these infections facilitates growth of antibiotic resistant urinary pathogens such as ESBLs-producing bacteria, which imposes substantial burden on medical cost due to increased risk of worse outcomes of UTIs caused by these bacteria. Post-menopausal women found to have much higher risk of T2DM-UTIs because drop in estrogen level is associated with insulin resistance and faulty regulation of metabolic homeostasis^13^. Majority of T2DM patients with UTIs in the present study (69%) were females, and the highest percentage of them (47.4%) were in post-menopausal period (55–65 years). This agreed with previous studies done by Zubair et al.,^14^ and Patra et al. from India,^15^ On the contrary, Mogaka et al. (2018) from Kenya^16^, and Sibi et al.^17^ from India found that diabetic male were affected with UTIs more than women. Most of clean catch urine cultures from T2DM patients had significant bacteriuria (97.3%), this was in accordance with a study by Longdoh et al. 18 from Cameroon (81.6%). A high frequency of significant bacteriuria was found among female diabetic patients (70%), this was in agreement with study obtained by Patra et al.^15^ from India. There was a significant association between glucose concentration in urine and culture colony count, where 80.9% of urine samples with colony count ≥105 CFU/ml were cultured from T2DM patients with +3 glucosuria level (≥ 500mg\dl). This study was in accordance with findings by Selim et al. (2019)19 from Egypt who detected bacterial growth in 100% of urine samples from patients with +4 glucosuria level. Mono microbial etiology of UTIs was found to be more common in this study, where E.coli was the most prevalent organism (58.4%); similar findings had been reported by Zubair et al. (2019)^14^ from Pakistan and Rahim et al.^20^ from Bangladesh. Out of 103 UTIs caused by *E.coli* and *K. pneumoniae*, 25 (24.3%) were due to ESBLs producing isolates. These findings were comparable to findings reported by Onanuga et al. (2019) from Nigeria,^21^ Abayneh et al.^22^ from Ethiopia. In the present study more than two thirds (68%) of ESBLs producing isolates were *E. coli*; and similar finding been reported in several studies^22^–^24^. More than one fourth of *E. coli* (25.8%) and 21.6% of *K. pneumoniae* isolates were found to be ESBL- producers. Significant association between ESBLs positivity and advanced age, use of urinary catheters and misuse of antibiotics. This observation was parallel to other studies^25^–^27^. In the present study, ESBLs production appeared as a significant risk factor for acute kidney injury, where all patients with history of AKI had ESBL-UTIs (100%). Same finding were reported by Rahim et al. (2018) from Bangladesh^20^ and Ramadas et al. (2014) from New York^28^. The results of antimicrobial susceptibility revealed that E. coli showed high resistance percentages to ampicillin (78.8%), followed by cefaclor (63.6%), norfloxacin (57.6%) and nitrofurantoin (54.6%). Zubair et al. (2019) showed similar resistance percentages to cefaclor, norfloxacin and nitrofurantoin (50%, 62.7% and 56.8%, respectively)^14^.

*K. pneumoniae* isolates showed high resistance percentages to nitrofurantoin (70.3%), cefaclor (67.6%) and norfloxacin (51.4%). This high level of resistance to norfloxacin and nitrofurantoin could be explained by absence of strict antibiotics use policy in the community, added to the exponential increase in prescription of these antibiotics for empiric treatment of hospitalized patients with positive urine cultures. The antimicrobial profile of ESBLs producing isolates showed significantly higher resistanceo aztreonam, all cephalosporin, and amoxicillin clavulanate compared to non-ESBLs producing isolates.

blaTEM was found to be the predominant gene (59.1%) in ESBL isolates, this finding was consistent with those found by Fattouh et al.^29^ from Egypt. blaCTX-M gene was not detected in any of tested isolates. Most of E. coli isolates harbored blaTEM gene (68.8%), while half of tested K. pneumoniae isolates had blaSHV gene. Plasmid-encoded ESBLs-producing bacteria can show co-resistance to quinolones, aminoglycosides and sulfonamide. In the present study 69.8% and 38.5% of isolates harboring blaTEM gene were found to be resistant to norfloxacin and amikacin, respectively. Isolates harboring blaSHV showed lower resistant rates to norfloxacin and nitrofurantoin (33.3%) and full sensitivity to amikacin. In the present study, the observed high level of resistance to imipenem (46.2%) among blaTEM harboring isolates is worrisome, since carbapenems are the treatment of choice for these isolates. In the current study, three isolates that were phenotypically positive by DDST method lacked TEM, SHV, and/or CTX-M genes. This may be explained by that these three isolates may carry other ESBLs encoding genes, which coud not be detected by the used primers, or it could be chromosomally mediated AmpC β-lactamases. This finding was parallel to that reported by Bajpai et al.^30^ from India. However, Sowmiya et al. (2012) from India showed a 100% correlation between phenotypic and genotypic methods of ESBLs detection^31^.

## Figures and Tables

**Figure 1:**
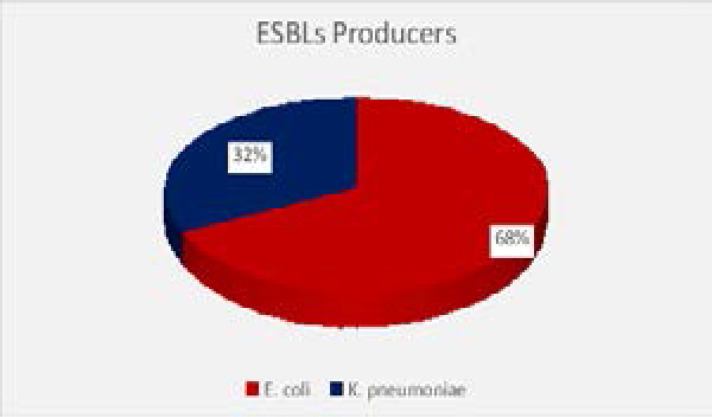
Distribution of ESBLs- producing urine isolates detected by DDST.

**Table 2:** Distribution of *E. coli* and *K. pneumoniae* isolates according to ESBLs production

ESBLs production	Isolates		Total
	*E. coli*	*K. pneumoniae*	
	No. (%)	No. (%)	No. (%)
**ESBL**	17 (25.8 %)	8 (21.6%)	25 (24.3%)
**Non ESBL**	49 (74.2%)	29 (78.4%)	78 (75.7%)
**Total**	66 (100%)	37 (100%)	103 (100%)
